# A review of the total syntheses of triptolide

**DOI:** 10.3762/bjoc.15.194

**Published:** 2019-08-22

**Authors:** Xiang Zhang, Zaozao Xiao, Hongtao Xu

**Affiliations:** 1 Department of Pharmacy, the First Affiliated Hospital of Zhengzhou University, Zhengzhou 450052, China; 2 College of Chemical Engineering and Materials Science, Tianjin University of Science & Technology, Tianjin 300457, China; 3 Shanghai Institute for Advanced Immunochemical Studies (SIAIS), ShanghaiTech University, Shanghai, 201210, China

**Keywords:** total synthesis, *Tripterygium wilfordii* Hook F, triptolide

## Abstract

Triptolide is a complex triepoxide diterpene natural product that has attracted considerable interest in the organic chemistry and medicinal chemistry societies due to its intriguing structural features and multiple promising biological activities. In this review, progress in the total syntheses of triptolide are systematically summarized. We hope to gain a better understanding of the field and provide constructive suggestions for future studies of triptolide.

## Introduction

**Figure 1:**
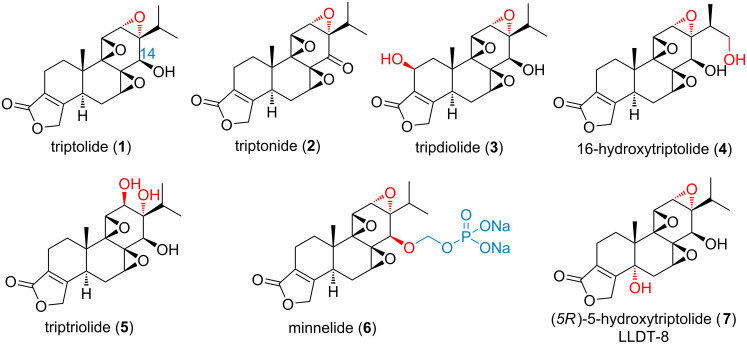
Structures of triptolide (**1**), triptonide (**2**), tripdiolide (**3**), 16-hydroxytriptolide (**4**), triptriolide (**5**), minnelide (**6**) and LLDT-8 (**7**).

Triptolide (**1**, Figure 1) is the first diterpenoid triepoxide isolated from *Tripterygium wilfordii* Hook F. (TWHF) in 1972 [1]. It has attracted an ever-increasing attention due to its intriguing structural features and various promising pharmacological activities [2–4], such as antiproliferative, antifertility [5], anti-osteoporosis [6], immunosuppressive and anti-inflammatory activities [7]. Reports have indicated that triptolide induces apoptosis, triggers autophagy [8], and arrests cell cycle progression through modulating the relevant signaling pathways involved in the regulation of reactive oxygen species (ROS) and/or nitric oxide (NO) [9], histone methyltransferase [10], HSP70 [11], Jak2, Bcl-2/Bax [12], caspase 8 [13], NF-κB [14], X-linked inhibitor of apoptosis protein (XIAP) [15], MAPK, PI3K [16], and MPK1, ERK-1/2, and JNK-1/2 [17]. The cross-talk network amongst these targets and signaling pathways are considered to be responsible for the multiple anticancer activities of triptolide [18–24]. Triptolide could also suppress inflammation and stimulate cytoprotection by regulating pro-inflammatory cytokines and chemokines such as IFN-γ [25], RANTES [26], IL-8 [27], COX-2 and NO [28]. These may associate with its effect on inflammatory relating diseases such as Parkinson’s disease [28], and kidney disease [29–30]. Meanwhile, through regulating immune-related cells, triptolide also showed great potential for the treatment of rheumatoid arthritis (RA) [31], systemic lupus erythematosus (SLE) and skin allograft rejection [32].

Despite the promising biological activities of triptolide, the narrow therapeutic window and multi-organ toxicity hindered greatly its clinical progress. Reports have shown that triptolide could cause reproductive toxicity [33], nephrotoxicity [34], hepatotoxicity [35], myocardial damage [36] and gastrointestinal tract symptoms [37]. In order to overcome the above issues and find derivatives with good drug-like properties, extensive total syntheses and structure modifications have been executed in the past two decades [38–51]. With the increasingly clear structure–activity relationships (SARs) [52–61], some derivatives of triptolide, such as minnelide **6** and (5*R*)-5-hydroxytriptolide (LLDT-8, **7**) have progressed into clinic for the treatment of pancreatic cancer and rheumatoid arthritis (RA) [61–62], respectively.

**Figure 2:**
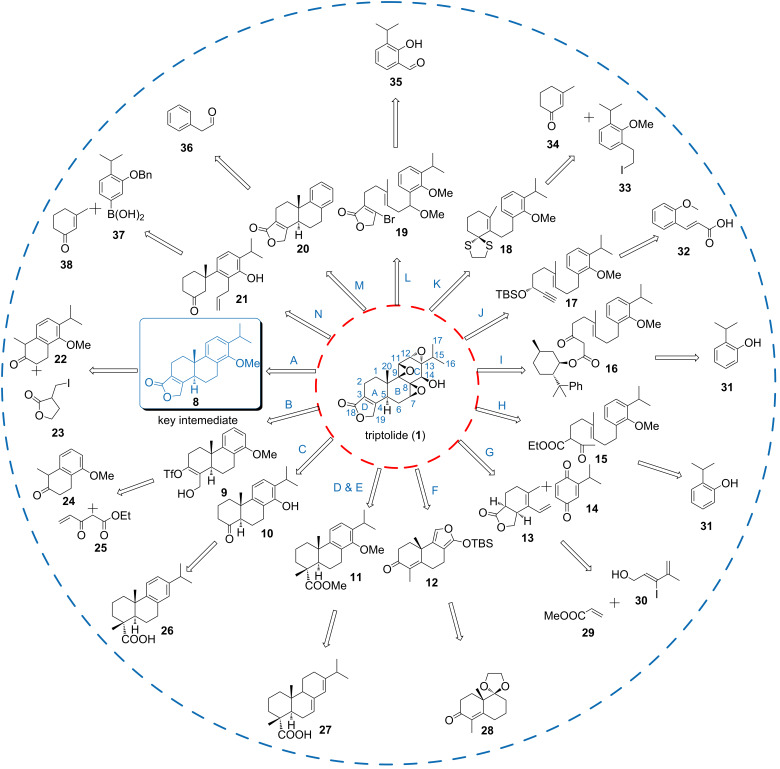
Syntheses of triptolide.

In order to gain a comprehensive and deep understanding of the area and provide suggestions for triptolide’s future studies, the recent progress of the total syntheses was systematically reviewed in this article. Syntheses were clustered based on: i) syntheses using tetralone (Figure 2, route A nad B), ʟ-abietic acid and/or ʟ-dehydroabietic acid as starting materials (route C, D and E); ii) syntheses using Diels–Alder reactions for the construction the A, B and C-rings (route F and G); iii) syntheses using polyene cyclization to construct the core structure (route H, I, J, K, and L); iv) syntheses highlight the utilization of metal-catalyzed reactions (M and N).

## Review

The intriguing structural features of triptolide and its relatives have provided a rich playing field for the design and development of total synthesis strategies. Structurally, it has nine chiral centers, three successive epoxides and a butenolide. Since the pioneering works of Berchtold, Tahara and their co-workers [45,63–65], several racemic and/or asymmetric total syntheses of triptolide and its relatives have been carried out in many research groups.

**Scheme 1:**
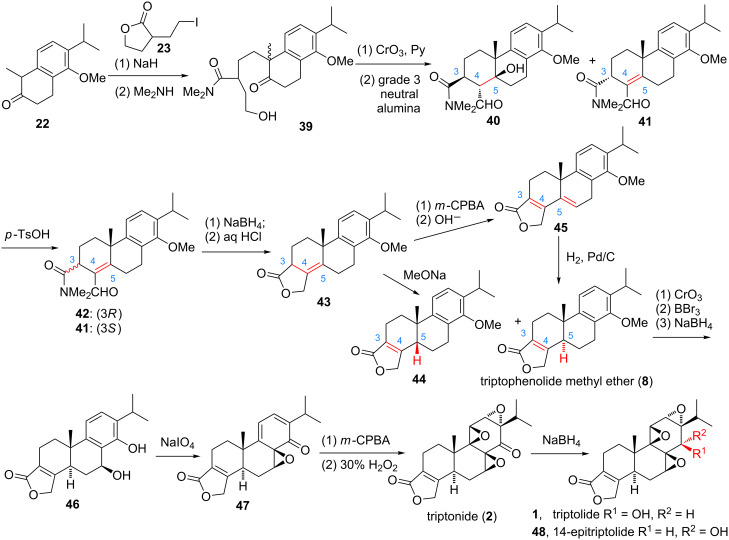
Berchtold’s synthesis of triptolide.

The first racemic total synthesis of triptolide was reported 1980 by Berchtold and co-workers (Figure 2, route A, and Scheme 1) [66]. The key steps include: i) construction of the A-ring by aldol condensation, ii) construction of the butenolide (D-ring) by acid-catalyzed lactonization, and iii) construction of the epoxides by a newly developed methodology. The synthesis commenced from the alkylation of tetralone **22** with 3-(2-iodoethyl)dihydrofuran-2(3*H*)-one (**23**) to give diastereomeric lactones that subsequently reacted with dimethylamine to afford a 1:1 mixture of diastereomeric amides **39**. Collins oxidation of **39** gave an aldehyde intermediate, which subsequently subjected to an aldol condensation using a ten-fold weight excess of neutral alumina quantitatively provided **40** and **41** in a small-scale reaction. The yield varied in large-scale reactions mainly due to the difficulty in extraction of **40** and **41** from the large quantity of alumina. Dehydration of the mixture of **40** and **41** in benzene quantitatively afforded a 1:2 mixture of **42** and its C-3 epimer **41**. Reduction of the epimers with sodium borohydride and subsequent treatment with hydrochloric acid (2 N) gave single isomer **43**. Treatment of **43** with methoxide ions in methanol at room temperature for 15 min gave the desired C-5 *trans*-butenolide **8** (40%) along with its C-5 *cis*-epimer **44** (60%), which is the sole product from the base-catalyzed isomerization of **43**. Epoxidation of **43** gave a C-4,5-epoxide intermediate, which was isomerized in the presence of base and dehydrated to give diene **45**. Reduction of **45** with 10% Pd/C afforded **8** in good yield (60%) after recrystallization. Benzylic oxidation of **8** (CrO_3_/HOAc, 45%), followed by C-14 ether cleavage (BBr_3_) and subsequent sodium borohydride reduction afforded **46** with the desired stereochemistry of the C-7 benzylic hydroxy group. Compound **46** was converted to triptonide **2** by Alder periodate reaction (NaIO_4_, 74%), and a sequencing *m*-CPBA epoxidation and basic hydrogen peroxide oxidation (H_2_O_2_/OH^−^) procedure (two steps, 28%). Finally, sodium borohydride reduction of **2** afforded triptolide (**1**, 21%) and 14-epitriptolide (**48**, 68%). Overall, the first total synthesis of racemic triptolide was finished from tetralone **22** in 16 steps. Although the intractable problems in some transformations, such as the neutral alumina-mediated aldol condensation to produce **40** and **41**, the isomerization of olefin **43**, the benzylic oxidation of **8**, the use of *m*-CPBA to introduce the C-9,11 epoxide and the non-stereoselective reduction of the C-14 carbonyl group using sodium borohydride, caused an unacceptable overall yield (1.6%). This pioneering work undoubtedly established the basis for the future syntheses of triptolide.

**Scheme 2:**
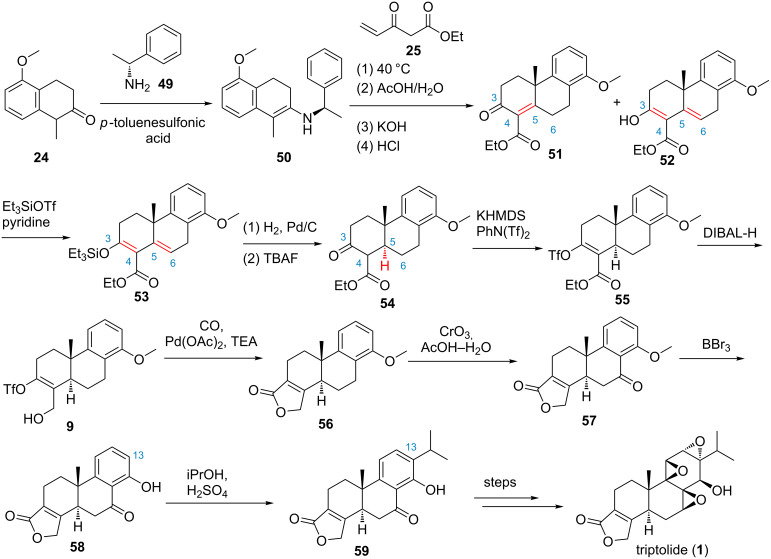
Li’s formal synthesis of triptolide.

In 2014, Li and co-workers further reported a formal asymmetric synthesis of triptolide from tetralone **24** (Figure 2, route B and Scheme 2) [50], featuring a Robinson annulation of Nazarov’s reagent **25** with 5-methoxy-2-tetralone **24** in the presence of enantiomerically pure (*R*)-α-phenylethylamine (**49**) to generate key tricyclic intermediates **51** and **52**, a Pd(II)-catalyzed carbonylation–lactonization reaction of **9** to construct the butenolide (D-ring), and a Friedel–Crafts isopropylation to install the C-13 isopropyl group. Still, the construction of the C-5 *trans* junction A-/B-ring was problematic, direct reduction of **51** and **52** with either Pd/BaSO_4_/H_2_ or Li/NH_3_/*t*-BuOH could not give the desired ketoester **54** in satisfactory yield for a target-oriented synthesis. Fortunately, after trying many conditions and procedures, a three-step indirect approach that include silyl ether formation of the mixture of **51** and **52**, subsequent Pd/C-catalyzed hydrogenation and tetrabutylammonium fluoride (TBAF)-mediated desilylation yielding the desired tricyclic **54** (83% yield, 98% ee). Overall, the known intermediate 7-oxotriptophenlide **59** was obtained in an efficient, elegant and scalable way in 10 steps with 18.5% overall yield. Importantly, by late-stage installation of the C-13 isopropyl group, this synthesis also provided a useful approach for the synthesis of other structurally relevant derivatives of triptolide such as C-15 and C-16 modified derivatives of triptolide.

**Scheme 3:**
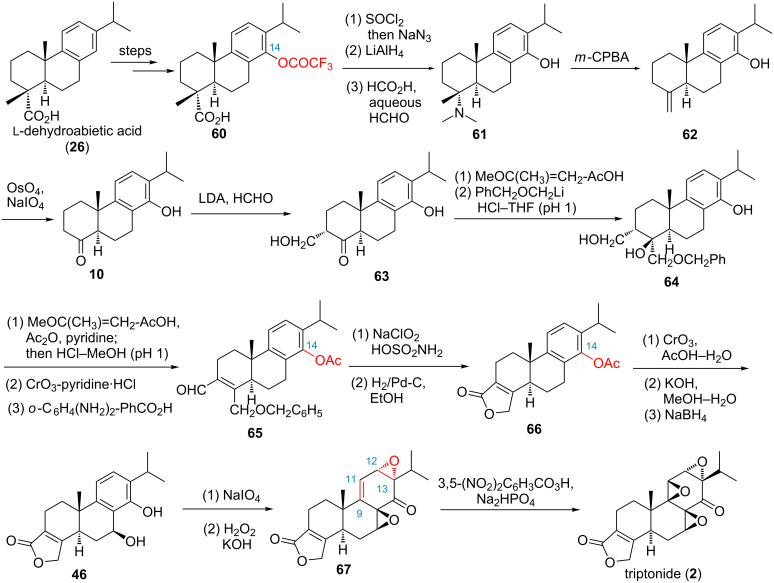
van Tamelen’s asymmetric synthesis of triptonide and triptolide.

In 1980, van Tamelen and co-workers reported an asymmetric synthesis of triptolide using readily available ʟ-dehydroabietic acid (**26**) as starting material (Figure 2, route C and Scheme 3) [67]. In this synthesis, the key step was the construction of the butenolide (D-ring). ʟ-Dehydroabietic acid was converted to C-14 trifluoroacetate **60** by a known electrophilic substitution procedure that was developed by Tahara and co-workers [64]. Curtius rearrangement of **60** gave an isocyanate intermediate, which was reduced with LiAlH_4_ followed by reductive amination affording tertiary amine intermediate **61**. Oxidation of **61** to its corresponding *N*-oxide followed by Cope elimination gave olefin **62**. Cleavage of olefin **62** with OsO_4_ and NaIO_4_ afforded ketone intermediate **10**, which was enolized by (iPr)_2_NLi (LDA) and further reacted with formaldehyde to afford hydroxy ketone **63**. Protection of the hydroxy group of **63** as 2-methoxypropyl ether, followed by successive treatment with PhCH_2_OCH_2_Li and HCl–THF (pH 1) gave triolmonobenzyl ether **64**. Protection of the phenolic hydroxy group to its corresponding monoacetate followed by oxidation of the primary hydroxy group and dehydration yielded α,β-unsaturated aldehyde **65**. Oxidation of **65** to the corresponding carboxylic acid followed by hydrogenolysis with H_2_/Pd-C led in spontaneous lactonization to give the key butenolide **66**. Oxidation of **66** with CrO_3_/AcOH–H_2_O, followed by saponification and reduction afforded known benzyl alcohol **46** (19% from **66**). Then, phenol **46** was converted to the corresponding epoxydienone via the methodology developed by Alder et al., which was treated without purification with basic H_2_O_2_ to yield diepoxide **67**, along with its 12,13-β-isomer. The mixture was immediately oxidized with 3,5-(NO_2_)_2_C_6_H_3_CO_3_H and Na_2_HPO_4_ to give triptonide (**2**, 15% from **67**), and reduction of triptonide via the reported procedure finalized the synthesis of triptolide. Overall, the first asymmetric total synthesis of (−)-triptonide (19 steps, 0.06% yield) and formal synthesis of triptolide were realized from ʟ-dehydroabietic acid (**26**). Although the overall yield of the synthesis is very low, the authors can ensure the optical purity of tripolide by utilizing the natural building block ʟ-dehydroabietic acid, and therefore could give a lot of inspiration for the future syntheses of triptolide and other related natural products from resource-abundant natural scaffolds.

Alternatively, inspired by the use of a natural diterpene scaffold as starting material for the synthesis of triptolide by van Tamelen and Tahara, Alvarez-Manzaneda’s group and Li’s group, respectively, reported formal syntheses of triptolide from ʟ-abietic acid (**27**, Figure 2, route D and E) [68–69]. In both syntheses, the key steps include the regioselective dihydroxylation and etherification to introduce the C-14 hydroxy group and the construction of the butenolide moiety. Particularly, the characteristics of Li’s route are low cost, high yield (9 steps, 44% yield) and easy handling (all intermediates could be scaled up to 100 grams without losing of yield) for the synthesis of key intermediate **8**.

**Scheme 4:**
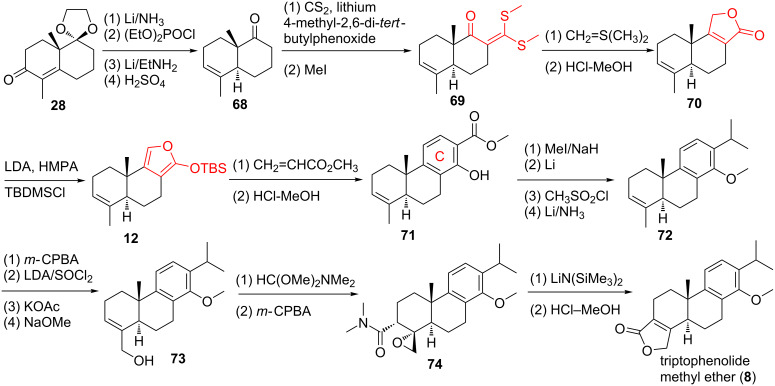
Van Tamelen’s (method II) formal synthesis of triptolide.

In order to further improve the synthesis of the key intermediate triptophenolide methyl ether (**8**), van Tamelen and co-workers further developed a synthesis as shown in Scheme 4 (Figure 2, route F) [70]. This synthesis features two new methodologies of butenolide formation. The first butenolide formation started with the reaction of ketone **68** with carbon disulfide (CS_2_) and iodomethane (MeI) to give the ketene dithioacetal intermediate **69**, which was subjected to a Corey–Chaykovsky epoxidation, followed by acid hydrolysis to give butenolide **70**. The second one is the reaction of alicyclic alcohol **73** with dimethylformamide dimethylacetal to give an allylic amide by means of a [2,3]-sigmatropic rearrangement of a carbene intermediate. Epoxidation the allylic amide with *m*-CPBA gave **74**, followed by lithium hexamethyldisilazide-induced β-elimination and acid hydrolysis to give the key triptophenolide methyl ether (**8**) in racemic form (16.5%).

**Scheme 5:**
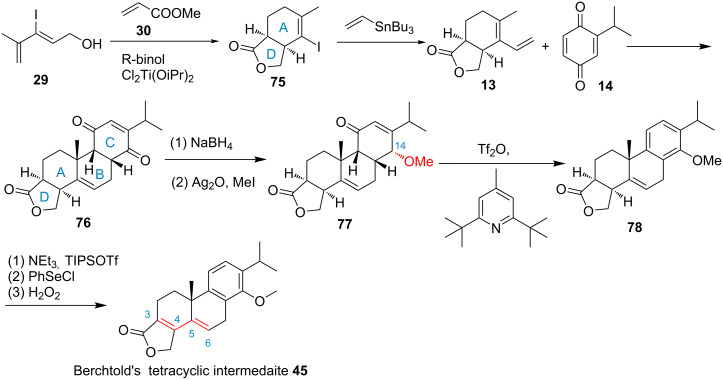
Sherburn’s formal synthesis of triptolide.

In 2008, Sherburn and co-workers developed an approach to the formal synthesis of triptolide (Figure 2, route G, Scheme 5) [71]. Key features of the synthesis include two intermolecular Diels–Alder reactions and a newly developed deoxygenative aromatization procedure. The first enantioselective Diels–Alder reaction, which is an intermolecular cycloaddition and lactonization between (*Z*)-3-iodo-4-methylpenta-2,4-dien-1-ol (**29**) and methyl acrylate (**30**) in the presence of Mikami’s (binol)TiCl_2_ catalyst to form the A- and D-ring. The second one involves the reaction of the bicyclic intermediate **13** and 2-isopropyl-1,4-benzoquinone (**14**) to form the B- and C-ring. Finally, a regio- and stereoselective reduction, methylation and dehydration procedure and a selenylation, oxidation and elimination procedure were employed to successfully achieve Berchtold’s tetracyclic C-5,C-6 olefin intermediate **45** in only 7 steps with 8.1% overall yield in a protecting-group-free synthesis. However, we know the conversion of Berchtold’s C-5,C-6 tetracyclic olefin intermediate **45** to triptophenolide methyl ether (**8**) is a troublesome work (60% yield) due to the formation of the thermodynamic more stable C-5 *cis*-epimer **44** [66]. Thus, it may not be possible to obtain the desired triptophenolide methyl ether (**8**) in a satisfactory yield for a target-oriented synthesis.

**Scheme 6:**
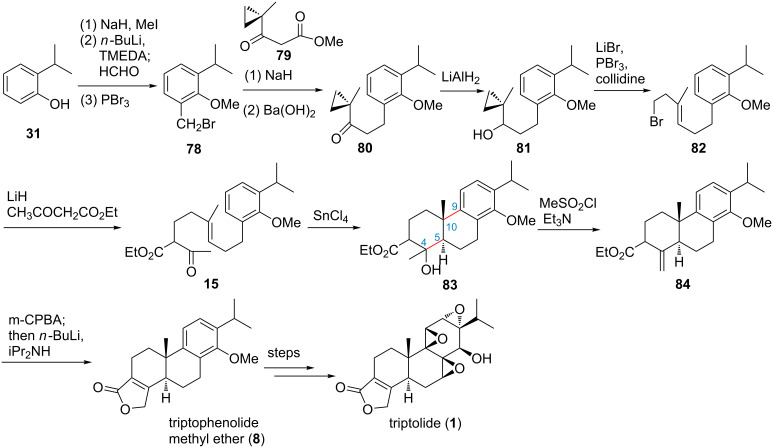
van Tamelen’s biogenetic type total synthesis of triptolide.

As the key structure element of various di- and triterpenes, the *trans-*decalin scaffold is the ideal objective of numerous methodology studies. Inspired by nature’s highly efficient and stereochemically controlled syntheses of terpenes, up to now, various synthetic strategies to *trans*-decalin have been developed, e.g., Brønsted acid or Lewis acid-mediated cationic polyene cyclization, transition-metal- or photocatalyst-mediated radical polyene cyclization [72]. The key to such transformation is to install a proper initiator within the substrate such as an allylic alcohol, an acetal, an aziridine, an *N*-acetal, a hydroxylactam, or a 1,3-dicarbonyl moiety.

van Tamelen and co-workers originally reported a nature-inspired acid-induced cationic polyene cyclization to construct the key *trans-*decalin scaffold (A- and B-ring) of triptolide (Figure 2, Scheme 6 and route H) [73]. In their synthesis, 2-isopropylphenol (**31**) was used as starting material to construct the key cyclization precursor ketoester **15**, which was cyclized in the presence of SnCl_4_ to give tricyclic intermediate **83**, followed by steps of functional group modification to install the butenolide (D-ring), and to finish the racemic synthesis of the key intermediate triptophenolide methyl ether (**8**) in 12 steps with 15% yield. Interestingly, in this synthesis only four intermediates needed to be purified.

**Scheme 7:**
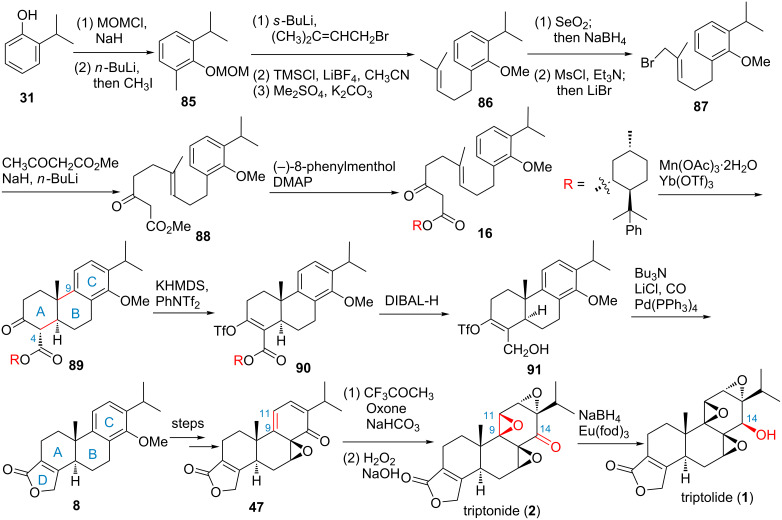
Yang’s total synthesis of triptolide.

In 1999, Yang and co-workers reported an elegant chiral auxiliary-assisted, lanthanide triflate-catalyzed oxidative radical polycyclization of olefin-cation-based enantioselective synthesis of triptolide (Figure 2, route I and Scheme 7) [74]. In this synthesis, commercially available 2-isopropylphenol (**31**) was used as starting material, protection of **31** with chloromethyl methyl ether (MOM), followed by ortho lithiation and methylation with iodomethane, provided intermediate **85**, which was lithiated and reacted with 3,3-dimethylallyl bromide, followed by changing the protecting group from MOM ether to methyl ether to provide olefin **86**. Allylic oxidation of **86** followed by nucleophilic bromination of the resulting allylic alcohol gave bromide **87**. Dianion displacement of the bromide of **87** gave ester **88**. Ester exchange of **88** with (+)-8-phenylmenthol gave the key cyclization precursor **16**. Oxidative radical cyclization of **16** in the presence of Mn(OAc)_3_ and Yb(OTf)_3_·H_2_O afforded the major tricyclic diastereomer **89** (dr = 38:1). Then the construction of the unsaturated lactone was performed by conversion of **89** to vinyl triflate **90**, followed by reduction and palladium-catalyzed carbonylation–lactone formation to give key intermediate **8** [75–77]. After that, the three successive epoxides were installed by known procedures with some modification. The highlights were the introduction of the second epoxide as a single diastereomer via in situ-generated methyl(trifluoromethyl)dioxirane and the reduction of the C-14 ketone in the presence of Eu(fod)_3_ to give triptolide (47%) together with its C-14 α-hydroxy epimer epi-triptolide (47%).

**Scheme 8:**
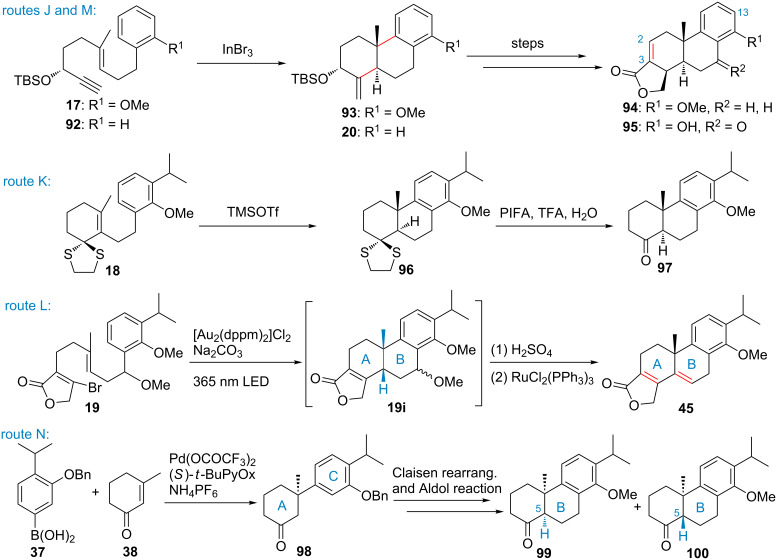
Key intermediates or transformations of routes J–N.

In 2014, Li’s group reported a divergent synthesis for triptolide and its relatives from commercially available acid **32** (Figure 2, route J and Scheme 8) [46]. This synthesis highlights the utilization of an indium(III)-catalyzed cationic polycyclization of **17** and a palladium-catalyzed carbonylation–in situ lactone formation to construct the key intermediate **94**, which could readily be converted to triptolide and its relatives such as triptophenolid, tripdiolide, and 16-hydroxytriptolide via palladium-catalyzed cross-coupling or Claisen rearrangement reactions. Importantly, by modification of the C-2,C3 olefin and late-stage installation of the C-13 isopropyl group, the synthesis also provides a new useful approach for the synthesis of other structurally relevant derivatives of triptolide.

In 2011, as their ongoing work that devoted to the synthesis of triptolide, Batti and co-workers developed a novel highly diastereoselective methodology that features *6-endo-trig* cyclization of 2-alkenyl-1,3-dithiolanes to access *trans*-decalins (Figure 2, route K) [78]. Density functional theory calculation (DFT) studies indicated that the 2-alkenyl-1,3-dithiolane moiety acts as a latent initiator, which triggers the cationic *6-endo-trig* cyclization in the presence of trimethylsilyl trifluoromethanesulfonate (TMSOTf) in a diastereoselective and stepwise manner. This novel methodology provides a shorter access to the intermediate **97**, which is a key intermediate for the synthesis of triptolide.

Recently, photoredox catalysis has emerged as a powerful and high-yielding method for the generation of carbon radicals via single-electron transfer (SET). In 2016, Barriault and co-workers reported a methodology that features the utilization of dimeric gold complex [Au_2_(dppm)_2_]Cl_2_ and ultraviolet A (UV, 365 nm) light to direct arylation of bromide-substituted butenolides or cyclic enones [51]. Photoexcitation (UV, 365 nm) of [Au-Au]^2+^ generated [Au-Au]^2+^*, which could then effectively reduce the vinyl C–Br bond via a SET to generate the vinyl radical. This radical then cyclizes with an aryl group to generated a tricyclic intermediate bearing a tertiary radical. A simultaneous oxidation of the tertiary radical and reduction of the [Au-Au]^3+^ ion could give the cyclization product and regenerate the dimeric gold photocatalyst. Later, the utility of this photoredox methodology was demonstrated in a concise formal synthesis of triptolide (**1**) via the reaction of bromobutenolide **19** under the optimal photoredox conditions to provide tetracyclic intermediate **19i** (dr = 1:1, Scheme 8, route L), with a *cis* A-/B-ring connection rather than the desired *trans* connection. Treatment of **19i** with H_2_SO_4_, followed by RuCl_2_(PPh_3_)_3_-catalyzed double bond isomerization gave the known intermediate **45**.

Thanks to the great advance of transition metal-mediated or catalyzed reactions that have been widely used for the construction of C–C, C–N, C–O, C–S, and C–X (X = F, Cl, Br or I) bonds in synthetic organic chemistry, and especially the transition metal-catalyzed functionalization of unreactive C–H bonds, either C_sp2_ or C_sp3_, a revolution in the synthesis of complex natural products (NPs) has been evolved. It provides a powerful toolbox to access complex natural structures and has the potential to streamline the synthesis. In 2014, Li and co-workers reported a metal-mediated reaction-based formal synthesis of triptolide and triptonide (Figure 2, route M) [48]. This synthesis highlights the use of Noyori’s ruthenium-catalyzed enantioselective transfer hydrogenation to introduce the chiral center; the indium(III)-catalyzed cationic polyene cyclization to construct the tricyclic A-, B- and C-ring system; palladium-catalyzed carbonylation and in situ lactone formation, and rhodium(II)-catalyzed double bond migration to construct the D-ring; and palladium-catalyzed C_sp2_–H oxygenation to install the C-14 hydroxy group. In 2016, Qin and co-workers developed a catalytic asymmetric route toward the formal synthesis of triptolide (Figure 2, route N) [43]. This synthesis highlighted the palladium-catalyzed asymmetric addition of arylboronic acid **37** to 3-methylcyclohex-2-en-1-one (**38**) to form the C-10 quaternary chiral center, and a subsequent Claisen rearrangement and an aldol reaction to furnish the *trans*-decalin A/B ring system.

## Conclusion and Future Perspectives

Taken together, the intriguing structural features, the promising multiple biological activities and the lack of natural sources have made triptolide an attractive target for total synthesis. So far, lots of total syntheses and formal syntheses of triptolide and its relatives have been established since the pioneering works of Berchtold and Tahara. Each synthesis has its advantages and disadvantages, the key points in the synthesis of triptolide are: i) the construction of the *trans*-decalin A-/B-ring system; ii) the construction of the butenolide (D-ring) and iii) the installation of the three successive epoxides (C-ring). In future syntheses the following issues should be considered: i) avoiding the isomerization of the C-5 chiral center; ii) development of a new method for the β-selective reduction of the C-14 carbonyl group of triptonide; iii) development of a new or improved synthesis to further satisfy the increasing demand of triptolide for the synthesis of clinical compounds such as LLDT-**8** and minnelide.

## References

[1] Kupchan S M, Court W A, Dailey R G, Gilmore C J, Bryan R F (1972). J Am Chem Soc.

[2] Liu Q (2011). Int Immunopharmacol.

[3] Ziaei S, Halaby R A (2016). Avicenna J Phytomed.

[4] Park B (2014). Biochimie.

[5] Yue Y, Hikim A P S, Wang C, Leung A, Baravarian S, Reutrakul V, Sangsawan R, Chaichana S, Swerdloff R S (1998). J Androl.

[6] Huang J, Zhou L, Wu H, Pavlos N, Chim S M, Liu Q, Zhao J, Xue W, Tan R X, Ye J (2015). Mol Cell Endocrinol.

[7] Qiu D, Kao P N (2003). Drugs R&D.

[8] Wei Y-m, Wang Y-h, Xue H-q, Luan Z-h, Liu B-w, Ren J-h (2019). Chin J Integr Med.

[9] Bao X, Cui J, Wu Y, Han X, Gao C, Hua Z, Shen P (2007). J Mol Med (Heidelberg, Ger).

[10] Zhao F, Chen Y, Li R, Liu Y, Wen L, Zhang C (2010). Toxicology.

[11] Phillips P A, Dudeja V, McCarroll J A, Borja-Cacho D, Dawra R K, Grizzle W E, Vickers S M, Saluja A K (2007). Cancer Res.

[12] Lin J, Chen L-Y, Lin Z-X, Zhao M-L (2007). J Int Med Res.

[13] YinJun L, Jie J, YunGui W (2005). Leuk Res.

[14] Zhu W, Hu H, Qiu P, Yan G (2009). Oncol Rep.

[15] Carter B Z, Mak D H, Schober W D, McQueen T, Harris D, Estrov Z, Evans R L, Andreeff M (2006). Blood.

[16] Miyata Y, Sato T, Ito A (2005). Biochem Biophys Res Commun.

[17] Tai C-J, Wu A T H, Chiou J-F, Jan H-J, Wei H-J, Hsu C-H, Lin C-T, Chiu W-T, Wu C-W, Lee H-M (2010). BMC Cancer.

[18] Hou Z-y, Tong X-p, Peng Y-b, Zhang B-k, Yan M (2018). Biomed Pharmacother.

[19] Liu L, Salnikov A V, Bauer N, Aleksandrowicz E, Labsch S, Nwaeburu C, Mattern J, Gladkich J, Schemmer P, Werner J (2014). Int J Cancer.

[20] Chen Z, Sangwan V, Banerjee S, Chugh R, Dudeja V, Vickers S M, Saluja A K (2014). Cancer Lett.

[21] Zhou G-S, Hu Z, Fang H-T, Zhang F-X, Pan X-F, Chen X-Q, Hu A-M, Xu L, Zhou G-B (2011). Leuk Res.

[22] Clawson K A, Borja-Cacho D, Antonoff M B, Saluja A K, Vickers S M (2010). J Surg Res.

[23] Carter B Z, Mak D H, Schober W D, Dietrich M F, Pinilla C, Vassilev L T, Reed J C, Andreeff M (2008). Blood.

[24] Raghavendra N M, Pingili D, Kadasi S, Mettu A, Prasad S V U M (2018). Eur J Med Chem.

[25] Krakauer T, Chen X, Howard O M Z, Young H A (2005). Immunopharmacol Immunotoxicol.

[26] Liu Q, Chen T, Chen G, Li N, Wang J, Ma P, Cao X (2006). Biochem Biophys Res Commun.

[27] Zhao G, Vaszar L T, Qiu D, Shi L, Kao P N (2000). Am J Physiol: Lung Cell Mol Physiol.

[28] Zhou H-F, Niu D-B, Xue B, Li F-Q, Liu X-Y, He Q-H, Wang X-H, Wang X-M (2003). NeuroReport.

[29] Sun M, Song H, Ye Y, Yang Q, Xu X, Zhu X, Zhang J, Shi S, Wang J, Liu Z (2019). Biomed Pharmacother.

[30] Chen D, Ma Y, Wang X, Yu S, Li L, Dai B, Mao Z, Liu H, Liu S, Mei C A J (2014). Kidney Dis.

[31] Fan D, Guo Q, Shen J, Zheng K, Lu C, Zhang G, Lu A, He X (2018). Int J Mol Sci.

[32] Yang S-X, Gao H-L, Xie S-S, Zhang W R, Long Z-Z (1992). Int J Immunopharmacol.

[33] Yang F, Ren L, Zhuo L, Ananda S, Liu L (2012). Exp Toxicol Pathol.

[34] Li J, Shen F, Guan C, Wang W, Sun X, Fu X, Huang M, Jin J, Huang Z (2014). PLoS One.

[35] Yang F, Wu L, Li Y, Wang D (2015). Drug Des, Dev Ther.

[36] Liu J, Jiang Z, Liu L, Zhang Y, Zhang S, Xiao J, Ma M, Zhang L (2011). Drug Chem Toxicol.

[37] Xu L, Qiu Y, Xu H, Ao W, Lam W, Yang X (2013). Food Chem Toxicol.

[38] Chen S-W, Zhou N-N, Li C (2012). Mini-Rev Org Chem.

[39] Xu H, Liu B (2019). Eur J Med Chem.

[40] Hou W, Liu B, Xu H (2019). Eur J Med Chem.

[41] Yang Y-q, Liang J, Han X-d, Tian R-m, Liu X-s, Mao W, Xu H-t, Liu B, Xu P (2019). Biomed Pharmacother.

[42] Yang Y-Q, Yan X-T, Wang K, Tian R-M, Lu Z-Y, Wu L-L, Xu H-T, Wu Y-S, Liu X-S, Mao W (2018). Front Pharmacol.

[43] Xu W-D, Li L-Q, Li M-M, Geng H-C, Qin H-B (2016). Nat Prod Bioprospect.

[44] Lai C K, Buckanin R S, Chen S J, Zimmerman D F, Sher F T, Berchtold G A (1982). J Org Chem.

[45] Sher F T, Berchtold G A (1977). J Org Chem.

[46] Xu H, Tang H, Feng H, Li Y (2014). J Org Chem.

[47] Xu H, Tang H, Yang Z, Feng H, Li Y (2014). Tetrahedron.

[48] Xu H, Tang H, Feng H, Li Y (2014). Tetrahedron Lett.

[49] Yang D, Wong M-K, Cheung K-K, Chan E W C, Xie Y (1997). Tetrahedron Lett.

[50] Zhang H, Li H, Xue J, Chen R, Li Y, Tang Y, Li C (2014). Org Biomol Chem.

[51] Cannillo A, Schwantje T R, Bégin M, Barabé F, Barriault L (2016). Org Lett.

[52] Zhou Z-L, Yang Y-X, Ding J, Li Y-C, Miao Z-H (2012). Nat Prod Rep.

[53] Kaloun E B, Long C, Molinier N, Brel V, Cantagrel F, Massiot G (2016). Tetrahedron Lett.

[54] Xu H, Tang H, Feng H, Li Y (2014). ChemMedChem.

[55] Xu H, Chen Y, Tang H, Feng H, Li Y (2014). Bioorg Med Chem Lett.

[56] Xu H, Tang H, Feng H, Li Y (2014). Eur J Med Chem.

[57] Xu H, Fan X, Zhang G, Liu X, Li Z, Li Y, Jiang B (2017). Biomed Pharmacother.

[58] Xu H, Liu L, Fan X, Zhang G, Li Y, Jiang B (2017). Bioorg Med Chem Lett.

[59] Aoyagi Y, Hitotsuyanagi Y, Hasuda T, Matsuyama S, Fukaya H, Takeya K, Aiyama R, Matsuzaki T, Hashimoto S (2008). Bioorg Med Chem Lett.

[60] Aoyagi Y, Hitotsuyanagi Y, Hasuda T, Fukaya H, Takeya K, Aiyama R, Matsuzaki T, Hashimoto S (2006). Bioorg Med Chem.

[61] Patil S, Lis L G, Schumacher R J, Norris B J, Morgan M L, Cuellar R A D, Blazar B R, Suryanarayanan R, Gurvich V J, Georg G I (2015). J Med Chem.

[62] Zhou R, Zhang F, He P-L, Zhou W-L, Wu Q-L, Xu J-Y, Zhou Y, Tang W, Li X-Y, Yang Y-F (2005). Int Immunopharmacol.

[63] Frieze D M, Berchtold G A, Blount J F (1978). Tetrahedron Lett.

[64] Tahara A, Akita H (1975). Chem Pharm Bull.

[65] Koike H, Tokoroyama T (1978). Tetrahedron Lett.

[66] Buckanin R S, Chen S J, Frieze D M, Sher F T, Berchtold G A (1980). J Am Chem Soc.

[67] Van Tamelen E E, Demers J P, Taylor E G, Koller K (1980). J Am Chem Soc.

[68] Alvarez-Manzaneda E, Chahboun R, Bentaleb F, Alvarez E, Escobar M A, Sad-Diki S, Cano M J, Messouri I (2007). Tetrahedron.

[69] Zhou B, Li X, Feng H, Li Y (2010). Tetrahedron.

[70] Garver L C, Van Tamelen E E (1982). J Am Chem Soc.

[71] Miller N A, Willis A C, Sherburn M S (2008). Chem Commun.

[72] Barrett A, Ma T-K, Mies T (2019). Synthesis.

[73] Van Tamelen E E, Leiden T M (1982). J Am Chem Soc.

[74] Yang D, Ye X-Y, Gu S, Xu M (1999). J Am Chem Soc.

[75] Yang D, Ye X-Y, Xu M (2000). J Org Chem.

[76] Yang D, Ye X-Y, Xu M, Pang K-W, Zou N, Letcher R M (1998). J Org Chem.

[77] Yang D, Xu M, Bian M-Y (2001). Org Lett.

[78] Goncalves S, Santoro S, Nicolas M, Wagner A, Maillos P, Himo F, Baati R (2011). J Org Chem.

